# Women speaker representation at SAGES annual meetings: a cross-sectional analysis

**DOI:** 10.1007/s00464-024-11034-z

**Published:** 2024-07-18

**Authors:** Sara M. Maskal, Jenny H. Chang, Varisha Essani, Ava Moe, Raha Al Marzooqi, Daphne Remulla, Hope T. Jackson, Lucas R. A. Beffa, Sharon S. Lum, R. Matthew Walsh, Ajita S. Prabhu

**Affiliations:** 1 Department of Surgery, Cleveland Clinic, 2049 E 100th St, Cleveland, OH 44195 USA; 2 Case Western Reserve University School of Medicine, Cleveland, OH USA; 3 University of North Carolina at Chapel Hill, Chapel Hill, NC USA; 4 Department of Surgery, University of Michigan Medicine, Ann Arbor, MI USA; 5 Department of Surgery, Loma Linda University School of Medicine, Loma Linda, CA USA

**Keywords:** Female surgeons, Gender diversity, Gender representation

## Abstract

**Background:**

Gender representation trends at the Society of American Gastrointestinal and Endoscopic Surgeons (SAGES) Annual Meetings and the effect of the 2018 ‘We R SAGES’ initiatives are unknown. We assessed gender trends in oral presentations at the SAGES Annual Meeting between 2012 and 2022 with a focus on assessing the impact of the 2018 initiatives.

**Methods:**

Abstracts selected for oral presentations from 2012 to 2022 were reviewed for presenter and first, second, and senior author gender. Gender was categorized as woman, man, or unknown using public professional profiles. Subsequent publications were identified using search engines. The primary outcome was the temporal trend of proportion of women in each role using interrupted time series analysis. Secondary outcomes included publication rates based on first and senior author genders in 2012–2018 versus 2019–2022.

**Results:**

1605 abstracts were reviewed. The proportion of women increased linearly in all categories: presenter (2.4%/year, *R*^2^ = 0.91), first author (2.4%/year, *R*^2^ = 0.90), senior author (2%/year, *R*^2^ = 0.65), and overall (2.2%, *R*^2^ = 0.91), (*p* < 0.01 for all). Prior to 2018, the proportion of women increased annually for presenters (coefficient: 0.026, 95% CI [0.016, 0.037], *p* = 0.002) and first authors (coefficient: 0.026, 95% CI [0.016, 0.037], *p* = 0.002), but there was no significant increase after 2018 (*p* > 0.05). Female second author proportion increased annually prior to 2018 (coefficient: 0.012, 95% CI [0.003, 0.021], *p* = 0.042) and increased by 0.139 (95% CI [0.070, 0.208], *p* = 0.006) in 2018. Annual female senior author proportion did not significantly change after 2018 (*p* > 0.05). 1198 (75.2%) abstracts led to publications. Women were as likely as men to be first (79% vs 77%, *p* = 0.284) or senior author (79% vs 77%, *p* = 0.702) in abstracts culminating in publications. There was no difference in woman first author publication rate before and after 2018 (80% vs 79%, *p* = 1.000), but woman senior author publication rate increased after 2018 (71% vs 83%, *p* = 0.032).

**Conclusion:**

There was an upward trend in women surgeons’ presentations and associated publications in the SAGES Annual Meetings over the last decade.

Despite gender parity in the proportion of women in general surgery residency, there are systematic differences in the proportion of women authorship on scientific articles which has real consequences in promotion and advancement [1–3]. While professional advancement is multifactorial, surgical societies, such as the Society of American Gastrointestinal and Endoscopic Surgeons (SAGES), play a key role in academic pursuits by selecting research for presentations and publications that in turn affects advancement of women surgeons and other groups that are historically underrepresented in surgery [4, 5]. Academic annual meetings are an opportunity to promote academic surgeons’ visibility through speakership and subsequent publication of original scientific work. One metric to assess improvement in gender diversity is increased participation of women as presenters at annual meetings.

SAGES was founded in 1981 and is now one of the premier societies in general surgery [6]. SAGES pledged their commitment to improving diversity and inclusion in 2018 with the ‘We R SAGES’ task force and SAGES climate survey publication. The task force specifically pledged to increase representation of women in committees, media content, and formal mentorship programs, and initiated a partnership with the Association for Women Surgeons [7]. The SAGES abstract review process is blinded, hence a proposed indirect metric for improvement in gender diversity is increased participation of women as presenters at annual meetings. The effect of this pledge on gender trends in oral presentations at the SAGES annual meeting is unknown. Evaluating the current progress of women surgeons’ representation at the annual meeting is crucial for understanding the society’s impact on gender parity in academic surgery.

The aim of our study was to evaluate trends in gender representation in oral presentations at the SAGES annual meeting before and after initiation of the ‘We R SAGES’ task force.

## Methods

After obtaining Institutional Review Board approval (Study Reference Number: 22-1038), SAGES meeting programs from 2012 to 2022 were reviewed for presenters, first authors, second authors, and senior authors of abstracts that were selected for oral presentation. This study was reported in accordance with the Strengthening the Reporting of Observational Studies in Epidemiology (STROBE) statement [8]. Investigators (SMM, JHC, AM, VE, RA, DR) utilized online search engines to identify publicly available professional profiles (i.e., institutional websites, Newsweek, LinkedIn, National Plan and Provider Enumeration System, etc.) associated with each abstract author’s name to categorize each individual’s gender as woman, man, or unknown based on gendered pronouns or other explicit gender statements. Gender was not assumed from name or image alone. If an individual was not identified, could not be linked to the listed institution, or if no gendered descriptors were found, then that author’s gender was marked as “unknown” to avoid introducing bias. Verified biomedical search engines (i.e., PubMed, Google Scholar, Scopus, etc.) were then used to identify subsequent publications from the abstract presented at SAGES. The first, second, and senior author of these publications were evaluated for gender as described above. SAGES membership data were provided by the organization.

The primary outcome of interest was the trend of proportion of women in each abstract authorship role over time which was evaluated using linear regression. A traditional interrupted time series analysis was used to investigate the effect of the ‘We R SAGES’ task force and the SAGES climate survey publication in 2018 on the proportion of women authors in each position over time [9]. Secondary outcomes included the gender proportions in each role, first and senior author gender concordance, publication rates based on gender of first and senior authors 2012–2018 compared to 2019–2022. Chi-square and *T*-tests were performed for descriptive analysis. All analyses were performed using R Software (4.3.1, Vienna).

## Results

A total of 1605 abstracts were available for review. In total, 30.4% (*n* = 484) of presenters, 30.7% (*n* = 489) of first authors, 28.9% (*n* = 460) of second authors, and 16.9% (*n* = 268) of senior authors were women. Gender was not categorized for 7.0% (*n* = 111) of presenters, 7.3% (*n* = 117) of first authors, 9.7% (*n* = 155) of second authors, and 6.0% (*n* = 96) of senior authors. The proportion of women increased linearly in all categories: presenter (2.4%/year, 95% CI [1.88, 2.99], *p* < 0.001, *R*^2^ = 0.91), first author (2.4%/year, 95% CI [1.84, 2.99], *p* < 0.001, *R*^2^ = 0.90), second author (2.0%/year, 95% CI [1.14, 2.85], *p* < 0.001, *R*^2^ = 0.72), senior author (1.9%/year, 95% CI [0.92, 2.85], *p* = 0.002, *R*^2^ = 0.65), and overall (2.2%/year, 95% CI [1.70, 2.66], *p* < 0.001, *R*^2^ = 0.91), over the entire study period. Women represented 13.1% of SAGES membership in 2012 compared to 23.3% in 2023 (1.1%/year, 95% CI [0.96, 1.32], *p* < 0.001, *R*^2^ = 0.95).

### Interrupted time series

**Fig. 1:**
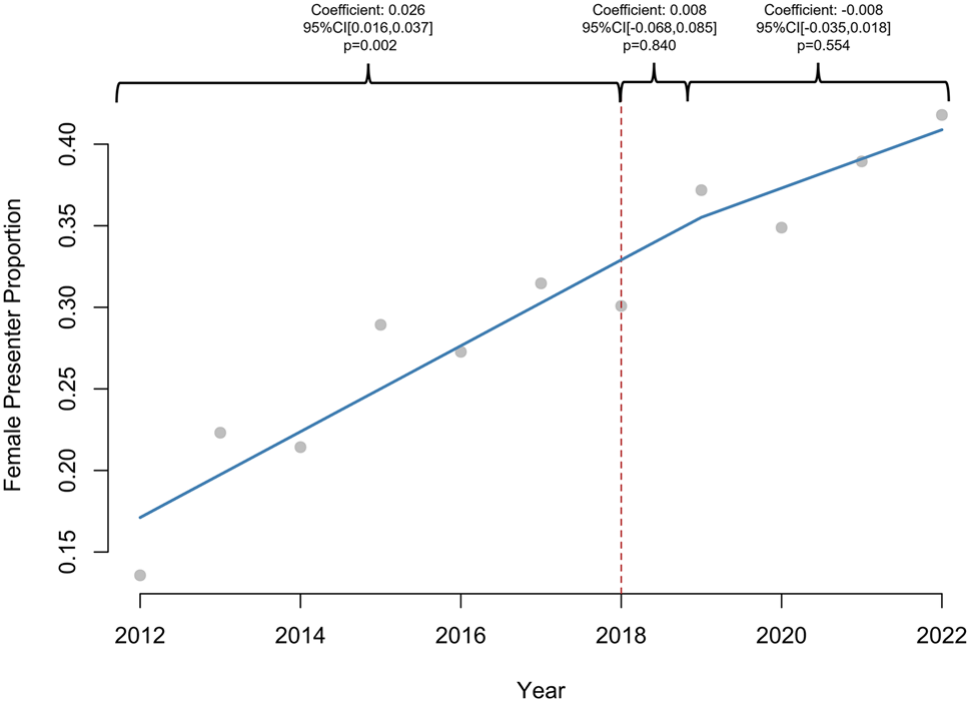
Interrupted time series analysis of women presenter proportion by year (2011–2022). *p-value < 0.05, **p-value < 0.01

**Fig. 2:**
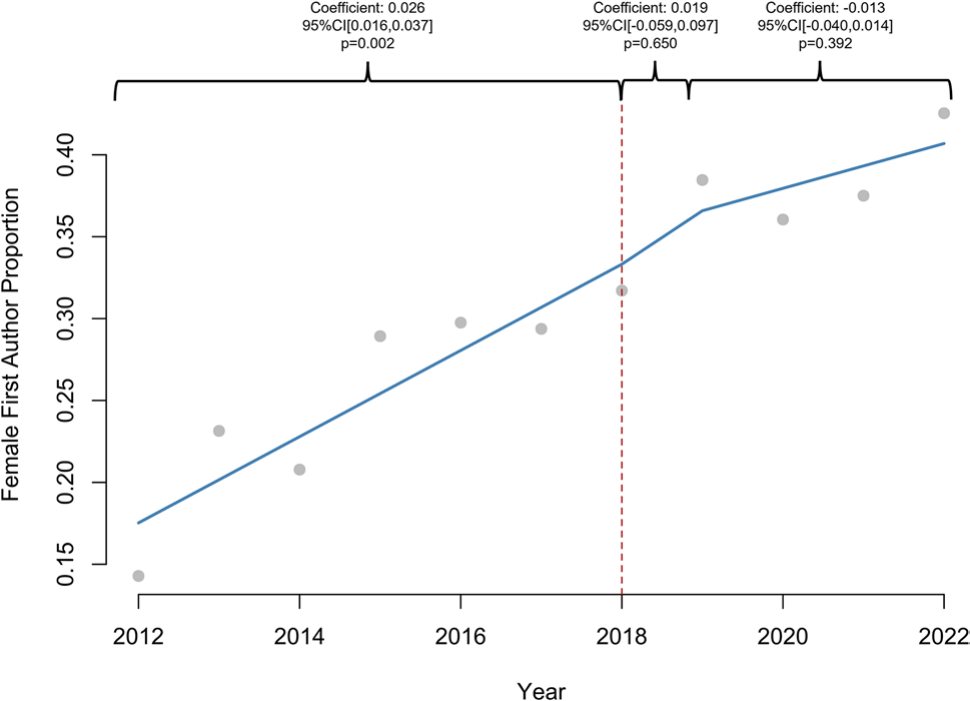
Interrupted time series analysis of women first author proportion by year (2011–2022). *p-value < 0.05, **p-value < 0.01

**Fig. 3:**
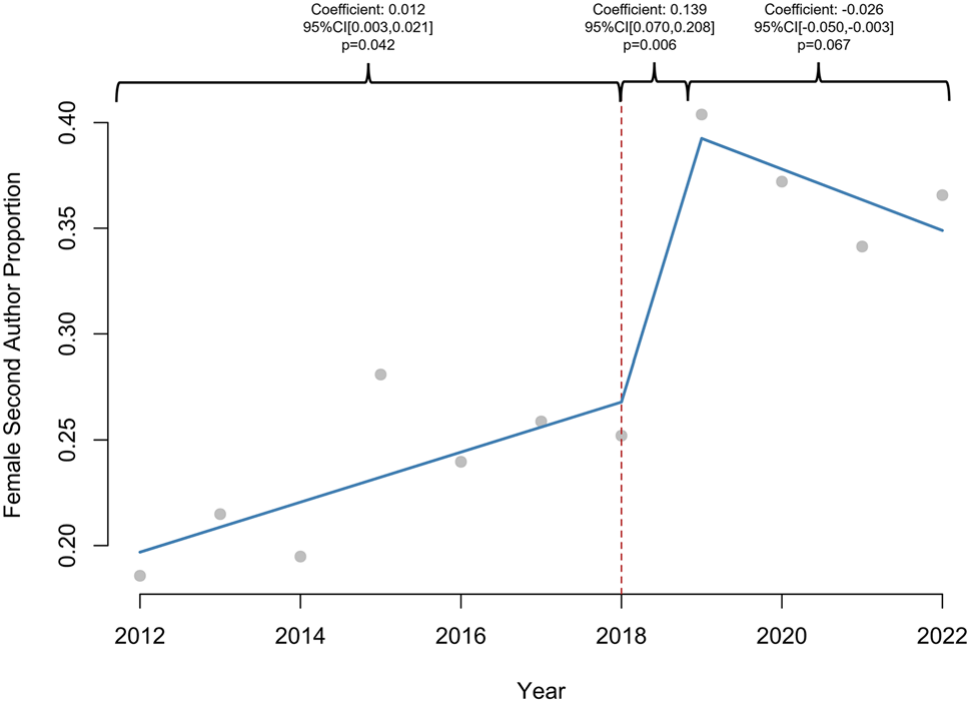
Interrupted time series analysis of women second author proportion by year (2011–2022). *p-value < 0.05, **p-value < 0.01

**Fig. 4:**
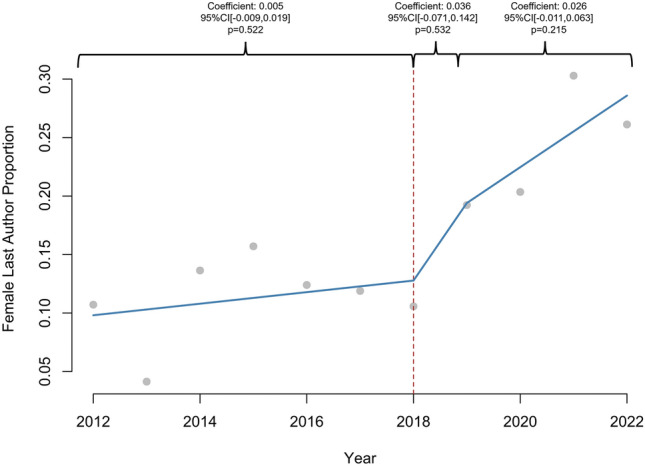
Interrupted time series analysis of women last author proportion by year (2011–2022). *p-value < 0.05, **p-value < 0.01

Prior to 2018, proportion of females increased annually for presenter (coefficient: 0.026, 95% CI [0.016, 0.037], *p* = 0.002), first author (coefficient: 0.026, 95% CI [0.016, 0.037], *p* = 0.002), and overall abstract authors (coefficient: 0.017, 95% CI [0.010, 0.025], *p* = 0.003), but there was no significant difference in 2018 or in the years following (*p* > 0.05) (Figs. 1, 2). The proportion of female second authors increased annually prior to 2018 (coefficient: 0.012, 95% CI [0.003, 0.021], *p* = 0.042) and increased by 0.14 (95% CI [0.070, 0.208]) in 2018 (*p* = 0.006) (Fig. 3). The annual proportion trend of female senior author did not significantly change over the course of the study period (*p* > 0.05) (Fig. 4).

### Publication outcomes

A total of 1198 (75.2%) abstracts led to publications, 1016 (84.8%) of which were published in *Surgical Endoscopy.* Overall, women were as likely as men to be first (79.0% vs 77.0%, *p* = 0.284) or senior author (79.0% vs 77.0%, *p* = 0.702) in abstracts culminating in publications. There was no difference in woman first author publication rates before and after 2018 (80.0% vs 79.0%, *p* = 1.000), but the senior author publication rate for women increased after 2018 (71.0% vs 83.0%, *p* = 0.032). Of abstracts with both first and senior author gender known, 64% (*n* = 905) were gender concordant. Gender concordance versus discordance between the first and senior authors did not show a difference in incidence of publication (78.5% vs 77.8%, *p* = 0.783).

## Discussion

In this cross-sectional analysis of the genders of authors of abstracts selected for oral presentations through blinded abstract review at SAGES between 2012 and 2022, positive trends for women presenters and authors emerged. The annual proportion of women second authors increased significantly in the year 2018. Although the changes made by ‘We R SAGES’ campaign in 2018 cannot be definitively credited with these trends across all authorship positions, there was an increase in first, second and senior authorship positions for women that outpaced the rate of increase in overall women membership in SAGES in the same period. It also outpaced the proportion of active US female surgeons which was 0.7%/year between 2010 and 2021.

In 2018, the ‘We R SAGES’ campaign published the results of a climate survey of the organization which demonstrated a high level of satisfaction, demonstrated by a mean satisfaction score of 8.1/10 and lower percentage of discrimination (6.4%). Specific themes that emerged in free text responses in the survey included a lack of diversity and dissatisfaction with transparent leadership position advancement. Pursuant to their findings, the campaign pledged to address these concerns with multiple initiatives including more transparent leadership selection, mentorship programs, collaborations with multiple societies representing women and other minority groups in surgery, image diversification, unconscious bias training for SAGES leadership, and proactively seeding diverse representation on committees and in session chairs and speakers at meetings [7]. One of the downstream effects that could be anticipated as a result of these efforts would be increased participation from women surgeons at annual meetings, which includes women trainees and mentors. The gender trends in abstract authorship over the past 11 years reflect a consistent, steady improvement in the representation of academic women surgeons consistent with the society’s efforts. Although the increase in women abstract authorship could not be causatively attributed to the ‘We R SAGES’ 2018 pledge to improving diversity, the upward trend is encouraging and intimates an inclusive culture.

Trends in women representation in authorship at SAGES compare favorably to other surgical society meetings. Our prior review of gender trends at a similar general surgery surgical society, Society of Surgery of the Alimentary Tract, revealed a slightly lower1.8% annual increase in women first author abstract publications and no increase in women senior authorship from 2010 to 2022 [10]. The increase in women authorship at the SAGES annual meeting is also higher than other surgical societies focused on general surgery [11, 12], endocrine surgery [13], and surgical education [14], but slightly lower in comparison to the American Society of Breast Surgeons [15]. While there is variation between societies, the percentage of women in speaking roles at annual meetings has increased steadily and suggests that women are improving their visibility in academic surgery on multiple frontiers [16]. Overall, documentation of progress made or lack thereof is necessary for transparent public reporting.

While there are many factors that may influence gender parity in academic achievement and career advancement, systemic barriers persist. Qualitative studies suggest that women surgeons encounter overt and implicit biases that hamper their engagement in academic departments [17]. Implicit association testing of surgeons demonstrated implicit and explicit biases associating women with family medicine and men with surgery [18]. These implicit biases shape social interactions and can negatively impact career prospects [19, 20]. Public statements of diversity and inclusion are a good initial step for academic surgical societies to start overcoming these barriers. Surgical societies that amplify messages of the importance of diversity in the surgical workforce helps to raise awareness and send a message to patients, surgical departments, and potential members that inclusion is a priority. With respect to the annual surgical society meetings, studies have shown that increasing women representation in meeting organization is associated with increased women participation [21]. While our data suggest that there was an increase in proportion women as second authors attributable to a change in 2018 but no other positions, it is important to note that women representation at the annual meeting was not a specified goal of the ‘We R SAGES’ campaign and therefore we are not evaluating the overall success of their campaign. Making conscious efforts to diversify the composition of committees, speakers, and leaders may help overcome the lack of representation for women and other groups that are historically underrepresented in surgery by expanding the general perception of who can be an expert in a field.

Our study has several limitations. As a retrospective, cross-sectional study, associations cannot be construed to mean causation. While upward trends were observed in multiple domains, this finding may be the reflection of increased participation of women in surgery and academic medicine, rather than being directly attributable to efforts by SAGES. Additionally, our data did not address all forms of representation. Specifically invited speakership as moderators and committee members are important avenues for representation, but that has been addressed in other recent publications [4, 16]. The unblinded process of inviting speakers is an opportunity for surgical societies to promote diverse representation at meetings. The small number of data points available for the time series analysis raises the possibility of a type two error however, this represents a limitation of the dataset and, as the society continues to collect this data, there remains opportunity to analyze larger periods of time. The total number of submitted abstracts as well as the gender composition of rejected abstracts was not available for comparison, which limits our ability to comment on whether the increased authorship of women was the result of increase submissions or selection processes. The frequency of individual authors was not captured, which would give an indication of whether the accepted abstracts represent of a small number of prolific authors or a larger number of unique individuals. Finally, we utilized public profiles to categorize individuals’ genders based on self-reporting. While acknowledging that individuals may choose to omit their pronouns, we found no examples of non-binary pronouns. Therefore, our definition of gender was limited to a binary definition for statistical analysis, but we acknowledge the importance of recognizing non-binary individuals as a traditionally underrepresented minority.

## Conclusion

As an organization, SAGES has demonstrated a positive increase in the representation of women surgeons in oral presentations at annual meetings. Intentional efforts to promote representation in academic societies are encouraging; however, the observed improvements in gender representation at SAGES over that past 11 years cannot be causatively attributed to the 2018 ‘We R SAGES’ initiative. We encourage surgical societies to prospectively track and evaluate demographic data to continuously evaluate and promote a diverse and inclusive culture.
